# To repair or not to repair: a case report of atrioventricular groove hematoma during mitral valve surgery

**DOI:** 10.1186/s13019-018-0828-0

**Published:** 2019-01-09

**Authors:** Hiroshi Nagamine, Yusuke Date, Takeshi Takagi, Yushi Kawase

**Affiliations:** Department of Thoracic and Cardiovascular Surgery, Yokohama Sakae Kyosai Hospital, 132 Katsura-cho, Sakae-ku, Yokohama, Kanagawa 247-8581 Japan

**Keywords:** Atrioventricular groove hematoma, Atrioventricular groove disruption, Left ventricular pseudoaneurysm, Transesophageal echocardiography

## Abstract

**Background:**

Atrioventricular groove hematomas during mitral valve surgery range from simple hematomas to complex atrioventricular disruptions that cause frank rupture with massive bleeding and subsequent mortality. A small or moderate-sized hematoma is reported to be present in the left atrioventricular groove in 10 to 30% of all patients immediately after mitral valve replacement. Despite the fact that atrioventricular groove hematomas are inherently unstable and unpredictable, conservative strategies are recommended due to the high mortality associated with additional surgical repair. Such conservative strategies, however, would not resolve the potential risk of rupture, and there also appears to be a certain degree of uncertainty to be overcome using the current advances in cardiac surgery.

**Case presentation:**

We present a case of atrioventricular hematoma during double valve replacement which was treated with conservative management. A left ventricular pseudoaneurysm developed after surgery, but spontaneously resolved completely within six months. After reflecting on our case, we developed a check sheet, including the anesthesiologist’s transesophageal echocardiography findings, for reasonable intraoperative decision-making regarding conservative management vs. additional surgical repair. Our check sheet helps organize the pathophysiological understanding of the injury and integrates partial findings from complementary viewpoints, and can be used to accurately assess intense situations and develop a common understanding among surgical team members.

**Conclusions:**

Our case involved an atrioventricular groove hematoma that occurred during mitral valve surgery and caused a left ventricular pseudoaneurysm. Conservative strategies yielded positive results. We hope our experience and original check sheet will be of value to surgical teams facing similar situations.

## Background

Atrioventricular groove (AVG) hematomas during mitral valve surgery range from simple hematomas to complex AVG disruptions that cause frank rupture with massive bleeding and subsequent mortality [1]. Kirklin’s textbook mentions that “a small or moderate-sized hematoma is present in the left AVG in 10% to 30% of all patients immediately after mitral valve replacement. If bleeding is not occurring, the patient’s condition remains good, and the hematoma does not increase in size, it should be left untreated and uninspected with nothing further done” [2]. Yet, because real-world clinical settings change by the minute and are often unstable and unpredictable, this conservative strategy that relies solely on a surgeon’s viewpoint appears to contain a certain degree of uncertainty to be overcome using the current advances in cardiac surgery. Here, we present a case of AVG hematoma during double valve replacement (DVR), which was treated with conservative management. A left ventricular pseudoaneurysm developed after surgery, but spontaneously resolved completely within six months. For reasonable intraoperative decision-making, we developed a check sheet that assesses real-time cardiac status and integrates partial findings from complementary viewpoints.

## Case presentation

A 64-year-old man, with severe multi-valvular disease detected during preoperative evaluation for colon diverticulitis, was referred for heart valve surgery. He had suffered recurrent life-threatening diverticular bleeding and accompanying heart failure. Transthoracic echocardiography (TTE) showed severe aortic regurgitation classified as type II (cusp prolapse) according to the functional classification developed by El Khoury et al., and severe mitral regurgitation caused by degenerative bi-leaflet prolapse with multiple eccentric regurgitant jets, exacerbated by secondary factors, including mitral annular and left ventricular (LV) dilation. Secure mitral repair with a shorter cardiopulmonary bypass (CPB) time would have been difficult due to the complex lesion; double valve replacement (DVR) was a safer and simpler procedure.

The anterior leaflet of the mitral valve was excised and its chordae cut at their insertion into the papillary muscles. The posterior leaflet and its subvalvular apparatus were preserved. A 29-mm bovine pericardial bioprosthesis was implanted in the intra-annular position and a 25-mm bovine pericardial bioprosthesis replaced the prolapsed aortic valve (Carpentier-Edwards Magna Mitral Ease Valve and Magna Ease Aortic Valve, respectively; Edwards Lifesciences; Irvine, CA, USA).

After DVR completion, extensive hematoma occurred surrounding the posterior atrioventricular groove during weaning from CPB. We suspected that deeply placed sutures around the posterior mitral annulus might have cut through the left ventricular wall. Full CPB support, aimed at a prompt reduction of the intraventricular pressure, was re-instituted immediately, and inotropic agents were tapered off to weaken the force of muscular contractions with the hope of suppressing the exacerbation of the injury. Routine intraoperative transesophageal echocardiography (TEE) monitoring was not utilized in this case; stable left ventricular contractility without bleeding was confirmed only by direct inspection before choosing conservative management. The re-weaning process was performed without any hemodynamic instability. The sternum was closed under stable conditions after administration of protamine sulfate.

**Fig. 1:**
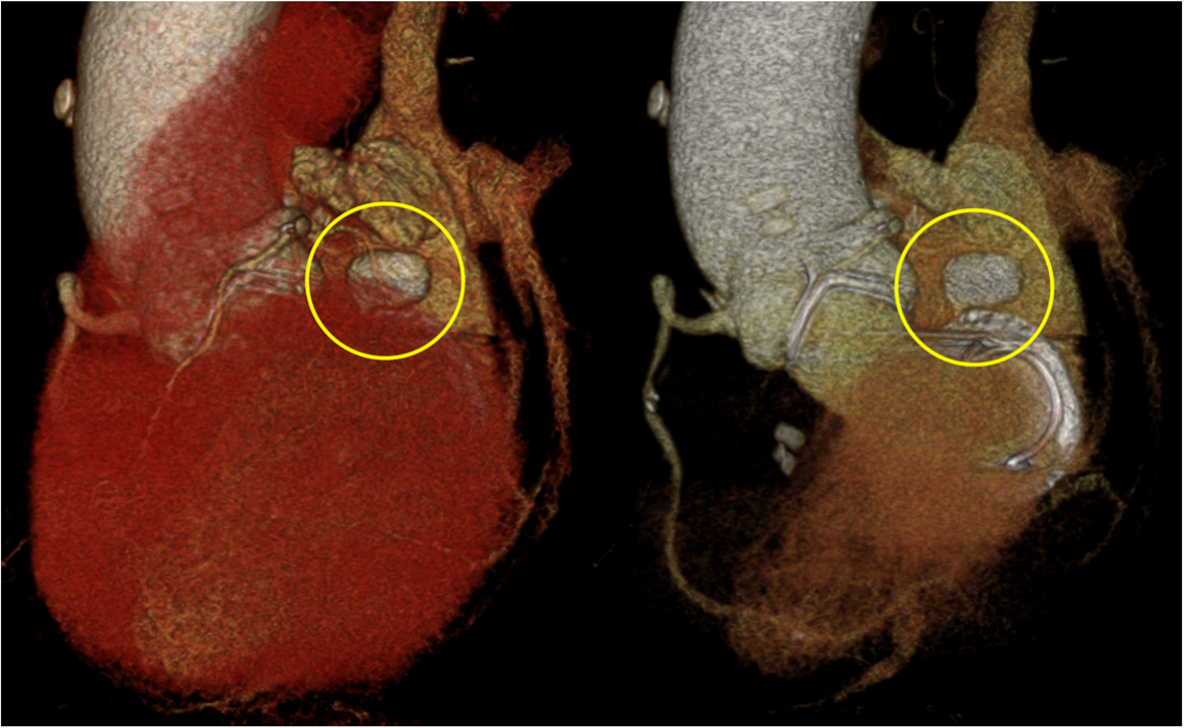
Left ventricular pseudoaneurysm following intraoperative atrioventricular groove hematoma. Volume rendering cardiac computed tomography on postoperative day 18 shows a pseudoaneurysm (20 × 12 mm) arising from the posterior wall of the LV just below the implanted mitral valve

The patient was taken into intensive care and extubated 12 h postoperatively. Postoperative routine TTE was unremarkable, whereas contrast-enhanced, electrocardiography (ECG)-gated, multi-detector computed tomography (CT) imaging on postoperative day 18 revealed a pseudoaneurysm (20 × 12 mm) arising from the posterior wall of the left ventricle just below the implanted mitral valve (Fig. 1). The fistulous tract of the pseudoaneurysm was 2 mm in diameter. We chose conservative management because the patient was asymptomatic, the pseudoaneurysm was not large, and the fistulous tract was narrow. After CT imaging confirmed aneurysm regression on postoperative day 32, the patient was discharged in good condition.

**Fig. 2:**
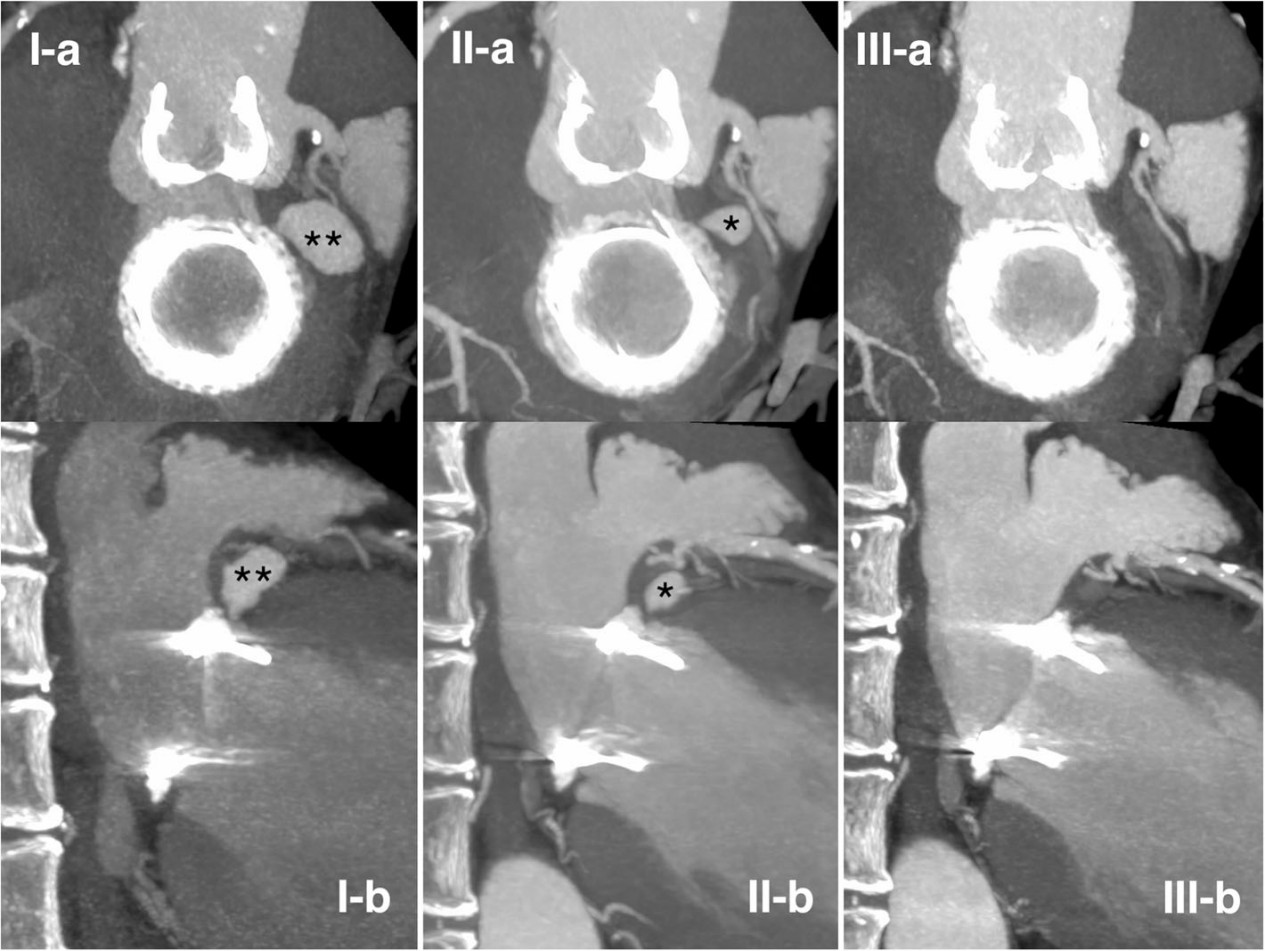
Pseudoaneurysm formation following atrioventricular groove hematoma. Changes over time are visualized by multiplanar reconstruction (MPR) of cardiac CT images. The upper row (I-a, II-a, and III-a) shows short-axis views and the lower row (I-b, II-b, and III-b) shows long-axis views. I-a and I-b: postoperative day 18, pseudoaneurysm and fistulous tract formation (**) arising from the posterior wall of the left ventricle just below the implanted mitral valve. II-a and II-b: three months postoperatively, regression in size of the pseudoaneurysm (*). III-a and III-b: six months postoperatively, complete resolution of the pseudoaneurysm

Six months postoperatively, the pseudoaneurysm had completely disappeared on CT imaging (Fig. 2). Three years after spontaneous resolution of the pseudoaneurysm, the patient has no cardiac complications, no recurrence of lower intestinal bleeding after colon surgery, and remains in good clinical condition.

## Discussion

Rupture of the posterior wall of the left ventricle following mitral valve surgery is a rare but potentially fatal complication [3]. This complication can be classified into three types based on the location of the tear [4]. Type I rupture occurs at the level of the AVG, which is the most common but most complex type and is also referred to as AVG disruption [5]. AVG disruption is typically accompanied by AVG hematoma, which is noticed on direct inspection by surgeons, occasionally as an early sign before frank rupture with massive bleeding and has a subsequent lethal clinical course [1, 6, 7].

AVG hematoma is usually detected at the termination of CPB when the heart starts beating again on its own [1, 6–8]. What is an acceptable upper size limit? Does uncertain hemostatic stability at one time point guarantee future safety? In practice, it is difficult to determine without hesitation whether to administer protamine sulphate to reverse anticoagulation and discontinue CPB, or perform an additional aortic cross-clamping procedure for restoration under cardioplegic arrest on the assumption that the situation will worsen.

**Fig. 3:**
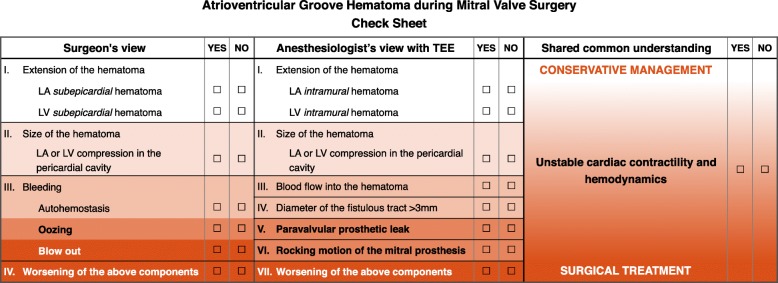
Original check sheet for intraoperative atrioventricular groove hematoma. The check sheet was designed for reasonable intraoperative decision-making based on two complementary viewpoints: the surgeon’s view of the surgical field and the anesthesiologist’s view using transesophageal echocardiography. Multidisciplinary severity stratification and a shared common understanding among surgical team members are essential when facing unstable and uncertain situations

With intraoperative TEE [1, 7, 8], the surgeon’s subjective decision based only on their restricted inspection might be obsolete. Instead, reliable judgement from an objective perspective using a multidisciplinary approach seems necessary, especially in potentially unstable and unpredictable situations. We developed a check sheet (Fig. 3), which includes the anesthesiologist’s TEE findings, after reflecting on our case for which routine monitoring should have been performed with intraoperative TEE. This check sheet helps organize the pathophysiological understanding of the injury and integrate partial findings from complementary viewpoints, and should be used to accurately assess intense situations and develop a common understanding among the surgical team. Conservative strategies, such as those found in Kirklin’s textbook, are recommended due to the high mortality associated with additional surgery [2]. On the other hand, additional surgical repair may become imperative to prevent rupture or control hemodynamic instability. An “internal” repair approach is most commonly recommended and consists of prosthesis explantation and complete AVG reconstruction, with a patch positioned from within the cardiac cavity [2, 4–6]. Our check sheet will be useful for reasonable intraoperative decision-making regarding conservative management vs. additional surgical repair.

AVG hematoma occasionally causes LV pseudoaneurysms in the chronic phase [2, 5]. Although 30–40% of untreated pseudoaneurysms rupture in the first year [9], the treatment strategy remains controversial [10–12]. Conservative management is possible if the pseudoaneurysm is small with a very narrow neck [10–12], but surgery is suggested for patients with large (i.e., ≥3 cm in diameter) or expanding false aneurysms [13]. In a recent report, Chenier et al. proposed that, for noninfectious pseudoaneurysms, surgery is only recommended for unstable patients, defined as a pseudoaneurysm ≥1 cm in size at initial presentation or rapid growth on serial imaging (> 0.5 cm in a 6-month period) [14]. In our case, a medium-sized pseudoaneurysm with a narrow fistulous tract was managed conservatively without surgical repair, and showed complete spontaneous resolution within six months.

## Conclusion

We presented a case of AVG hematoma that occurred during mitral valve surgery and caused left ventricular pseudoaneurysm. Conservative strategies yielded positive results in our case. We hope our experience and original check sheet designed for reasonable intraoperative decision-making are valuable for surgical team members facing similar situations.

## Abbreviations

AVG: Atrioventricular groove
CPB: Cardiopulmonary bypass
CT: Computed tomography
DVR: Double valve replacement
ECG: Electrocardiography
LV: Left ventricular
MV: Mitral valve
TEE: Transesophageal echocardiography
TTE: Transthoracic echocardiography
